# Characterizing the PRRSV nsp2 Deubiquitinase Reveals Dispensability of *Cis*-Activity for Replication and a Link of nsp2 to Inflammation Induction

**DOI:** 10.3390/v11100896

**Published:** 2019-09-26

**Authors:** Shaochuan Zhou, Xinna Ge, Can Kong, Teng Liu, Aijing Liu, Peng Gao, Jiangwei Song, Lei Zhou, Xin Guo, Jun Han, Hanchun Yang

**Affiliations:** Key Laboratory of Animal Epidemiology of the Ministry of Agriculture and Rural Affairs, College of Veterinary Medicine, China Agricultural University, Beijing 100193, China; 15210922708@163.com (S.Z.); gexn@cau.edu.cn (X.G.); kcaner@163.com (C.K.); Liuteng2018cau@163.com (T.L.); laj_91@126.com (A.L.); penggao@cau.edu.cn (P.G.); songjiangwei525@126.com (J.S.); leosj@cau.edu.cn (L.Z.); guoxin@cau.edu.cn (X.G.); yanghanchun1@cau.edu.cn (H.Y.)

**Keywords:** PRRSV, deubiquitinase, deubiquitination, *trans*-cleavage activity, *cis*-cleavage activity, inflammation

## Abstract

The papain-like cysteine protease 2 (PLP2) within the N-terminus of the porcine reproductive and respiratory syndrome virus (PRRSV) nsp2 replicase protein specifies a deubiquitinating enzyme (DUB), but its biochemical properties and the role in infection have remained poorly defined. By using in vitro assays, we found that the purified PLP2 could efficiently cleave K63 and K48 linked polyubiquitin chains Ub3-7 in vitro although displaying a differential activity in converting the respective ubiquitin dimers to monomer. The subsequent mutagenesis analyses revealed that the requirement for PLP2 DUB activity surprisingly resembled that for *cis*-cleavage activity, as several mutations (e.g., D91R, D85R, etc.) that largely ablated the DUB function also blocked the *cis*- but not *trans*-proteolytic cleavage of nsp2/3 polyprotein. Moreover, the analyses identified key mutations that could differentiate DUB from PLP2 *cis*- and *trans*-cleavage activities. Further reverse genetics analyses revealed the following findings: (i) mutations that largely blocked the DUB activity were all lethal to the virus, (ii) a point mutation T88G that selectively blocked the *cis*-cleavage activity of PLP2 did not affect viral viability in cell culture, and (iii) an E90Q mutation that did not affect either of the PLP2 activities led to rescue of WT-like virus but displayed significantly reduced ability to induce TNF-α production. Our findings support the possibility that the PLP2 DUB activity, but not *cis*-cleavage activity, is essential for PRRSV replication. The data also establish a strong link of nsp2 to pro-inflammatory cytokine induction during infection that operates in a manner independent of PLP2 DUB activity.

## 1. Introduction

Protein ubiquitination is an important post-translational modification that regulates a variety of cellular biological processes [1,2,3], i.e., signaling transduction [4], protein turnover [5], cell cycle progression [6], and the immune and inflammatory responses [4,7,8], etc. This modification, however, can often be reversed by deubiquitinases (DUBs) through removing various ubiquitin molecules from substrates [9]. Not surprisingly, many DNA and RNA viruses have evolved to encode DUBs to manipulate diverse cellular pathways [10]. The currently discovered viral DUBs largely resemble their cellular counterparts in structure and generally fall into two major classes: ubiquitin-specific proteases (USP) (e.g., coronaviruses, herpesviruses, picornaviruses, etc.) [11,12,13,14,15] and ovarian tumor proteases (OTU) (e.g., arteriviruses, nairoviruses and tymoviruses) [16,17,18,19,20,21,22,23]. Functionally, most of the viral DUBs have been demonstrated to exhibit an immune suppressive activity in vitro and negatively regulate host innate immunity such as interferon signaling and production of pro-inflammatory cytokines and chemokines [8,24], although the biological significance in virus infection remains to be determined for many of them. This report concerns the deubiquitinase of porcine reproductive and respiratory syndrome virus (PRRSV), a positive-stranded RNA virus in the family *Arteriviridae* of the order *Nidovirales* [25,26] and a major pathogen of swine that causes colossal annual economic losses to the worldwide swine industry [27,28,29,30,31].

**Figure 1:**
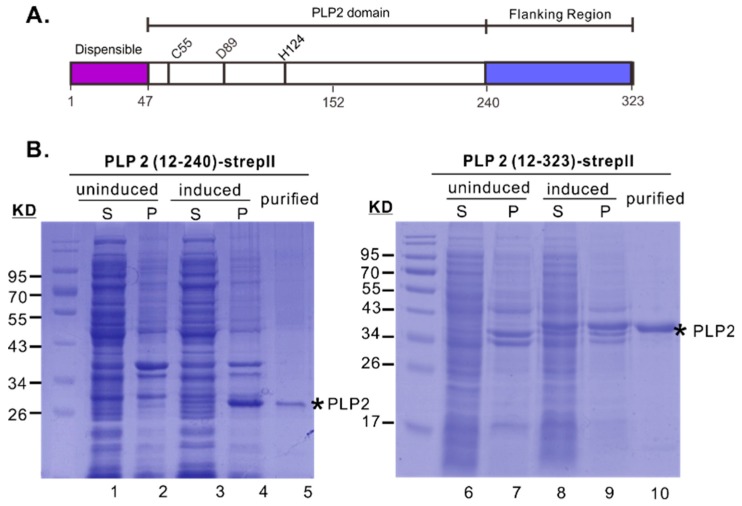
Expression, purification and DUB activity of PRRSV PLP2 domain. (**A**) Domain organization of the first 323 amino acids of PRRSV nsp2. C55-H124 are the putative catalytic dyad. (**B**) Expression and purification of PLP2 (12–323)-StrepII and PLP2 (12–240)-StrepII from *E. coli* BL21 cells. Abbreviations: S: supernatant, P: pellet.

The PRRSV deubiquitinase is specified by the papain-like protease 2 domain (PLP2) that is located within the N-terminus of nonstructural protein 2 (nsp2), a multi-domain and multi-functional replicase protein in the virus life cycle [20,21,32,33,34,35,36]. Phylogenetically, PRRSV PLP2 belongs to the newly identified OTU superfamily that represents a novel class of cysteine proteases [16], its putative catalytic sites (Cys55-His124) (Figure 1A) are highly conserved not only among arteriviruses but also among all OTU family members [16,37,38]. The core domain of PRRSV PLP2 has a size of about 100 amino acids (nsp2 aa. 47–150) based on the sequence alignment with the equine arteritis virus (EAV) PLP2 and other OTU family members [39,40]. Different from EAV PLP2, the downstream flanking sequence (aa. 152–240) of PRRSV PLP2 core (Figure 1A) is however required for the protease activity [19,34]. Earlier biochemical studies have demonstrated that the PRRSV PLP2 possesses both *cis*- and *trans*-cleavage activities that mediate the proteolytic cleavage at nsp2/3 junction [34]. Moreover, these two activities can be decoupled by a point mutation of the residue Asp-89 [34]. A D89N mutation can selectively block the *trans*- but not *cis*-cleavage activity of PLP2. Consequently, this mutation is lethal to PRRSV [34].

More recently, PRRSV PLP2 was shown to possess deubiquitinating activity in transfected 293 FT cells and can antagonize interferon signaling through inhibiting activation of NF-κB [17,20,21], a feature that is similar to the counterparts from EAV and nairoviruses of the family of *Bunyaviridae* [17,20,21]. Further, Deaton et al. have reported the DUB activity of PRRSV PLP2 by in vitro assay and found that the *E. coli* purified recombinant PLP2 (aa. 12–215) is able to cleave both K48 and K63 poly-ubiquitin chains in vitro [41]. On the other hand, although PRRSV PLP2 was shown to have deISGylation activity in transfected human cells [21], the recombinant PLP2 showed little in vitro deISGylating activity toward ISG15 of porcine origin [41,42], leaving in question whether it can actually efficiently cleave swine ISG15 conjugates in primary macrophages.

In any case, it is clear now that the PRRSV PLP2 possesses at least *cis*-, *trans*-cleavage, and DUB activities [20,34,41]. However, their biochemical properties and the contribution to viral infection have remained poorly defined. In this study, we started with characterizing the PLP2 DUB activity of HP-PRRSV strain JXwn06 by using in vitro and cell-based assays. By using site-directed mutagenesis, we were able to identify mutations that can differentiate the DUB activity from *cis*- and *trans*-cleavage activities and assess their roles in the context of PRRSV infection by reverse genetics analysis. Our results revealed novel biochemical aspects of PRRSV PLP2 and showed that the PLP2 DUB activity, but not the *cis*-cleavage activity, is likely important for PRRSV replication. Unexpectedly, we also revealed a strong link of PRRSV nsp2 to virus-induced inflammation that occurs in a DUB-independent manner. The findings further advance our understanding of PRRSV nsp2 function and it is the regulation of host immunity and has implications in antiviral drug and vaccine development.

## 2. Materials and Methods

### 2.1. Cells, Virus, and Antibodies

MARC-145 and HEK 293FT cells were maintained in Dulbecco’s modified Eagle’s medium (DMEM) (Invitrogen, Carlsbad, CA, USA) with 10% FBS (Invitrogen, CA, USA). Primary porcine pulmonary alveolar macrophages (PAMs) were obtained from specific-pathogen-free (SPF) pigs as previously described [43] and cultured in RPMI1640 medium (Invitrogen, CA, USA) containing 10% fetal bovine serum (FBS) (Invitrogen, CA, USA). The Chinese highly pathogenic PRRSV strain JXwn06 infectious cDNA clone was described previously [31].

Mouse anti-FLAG monoclonal antibody (F1804), mouse anti-HA monoclonal antibody (H3663), mouse anti-β-actin monoclonal antibody (A5441), and rabbit anti-Myc polyclonal antibody (C3956) were all purchased from Sigma-Aldrich (St. Louis, MO, USA). Horseradish peroxidase (HRP)-conjugated goat anti-mouse polyclonal antibodies and HRP-conjugated goat anti-rabbit polyclonal antibodies were purchased from ZSGB-Bio (Beijing, China). All the restriction enzymes used in this study were from New England Biolabs Inc. (Beverly, MA, USA).

### 2.2. Plasmid Construction

The prokaryotic expression plasmids expressing PLP2 (12–240)-strepII and PLP2 (12–232)-strep II were constructed by cloning the respective region coding for nsp2 aa. 12–240 and aa. 12–323 from HP-PRRSV strain JXwn06 (accession number: EF641008) into the vector pET-28a (Novagen, Madison, WI, USA) at the sites of Nco I and Xho I with a strep II tag attached to C-terminus for purification purpose. The mammalian plasmid expressing PLP2 (12–323)-myc was made by cloning the same PLP2 sequence into the vector pEGFP-N1 (Clonetech, CA, USA) at the sites of BamH I and Xho I in frame with c-myc epitope tag coding sequence. The plasmids pNsp2-Myc, pFlag-HA-Nsp2-3, and pFlag-HA-Nsp2-3Δ1–399 were generated by fusion expression of the indicated epitope tags with the corresponding coding region for nsp2, nsp2-3 or nsp2-3 lacking the first 399 amino acids in the vector pEGFP-N2 (Clonetech, Mountain View, CA, USA) at the sites of Bgl II and BamH I. All genes in the created eukaryotic plasmids are under the control of CMV promoter, and a Kozak core sequence is also included to allow optimal expression. For expression of the derivatives of PLP2, nsp2, or nsp2-3, the point mutation mutants were created by QuikChange site-directed mutagenesis. All the constructs were generated by standard recombinant DNA procedure and verified by DNA sequencing.

### 2.3. E. coli-Based Protein Expression and Purification

Bacterial strain BL21(DE3) containing the plasmid for PLP2 (12-323)-strepII or its derivative was cultured overnight at 37 °C and then inoculated at 1:1000 into 100 ml of yeast extract-tryptone medium culture (2XYT). When the bacterial density at 600 nm reached 0.6 to 0.7, protein expression was induced 32 °C for 7 h by addition of IPTG (Isopropyl-D-1-thiogalactopyranoside) (Sigma) at the final concentration 0.5 mM. The cells were harvested by centrifugation at 8000 rpm for 3–5 min and then resuspended in PBS with the protease inhibitor cocktail (Sigma, P8340). The cells were sonicated and lysed for 30 min at 4 °C on ice with 1% Triton X-100 (Sigma, T8787). The cell lysates were cleared by centrifugation at 12,000rpm for 5–10 min. The supernatants were incubated with 200 uL StrepTactin sepharose (GE healthcare, 45-002-414) at 4 °C overnight on the rocker. The StrepTactin sepharose beads were collected by centrifugation and washed six times with Tris buffer (20 mM Tris, pH 7.4). The target protein was eluted with 400 uL elution buffer (20 mM Tris, 2.5 mM desthiobiotin, 20% glycerol, pH 7.4).

### 2.4. In Vitro Fluorescent DUB Assay

The DUB activity of purified PRRSV PLP2 was assayed in 50 μL reaction containing indicated amount of purified PLP2 protein, 1 μM Ub-AMC (Boston biochem, U-550), and 1× reaction buffer (137 mM NaCl, 2.7 mM KCl, 4.3 mM Na2HPO4, 1.4 mM KH2PO4, and 2 mM DTT) in a 96-well black microplate. The fluorescent intensity (excitation, 345nm, emission, 445nm) of each well was observed by infinite M1000 Pro plate reader (Tecan, Inc).

### 2.5. In Vitro Cleavage of K48 and K64-Linked Polyubiquitin Chains

The DUB activity of purified PRRSV PLP2 was assayed in 20 μL reaction containing indicated amount of purified PLP2 protein, 2.5 μg K48- (Boston biochem, UC-220) or K63-linked polyubiquitin chains Ub3-7(Boston biochem, UC-320), and 1× reaction buffer (137 mM NaCl, 2.7 mM KCl, 4.3 mM Na2HPO4, 1.4 mM KH2PO4, and 2 mM DTT) in a 100 μL volume EP tube. The mixture was incubated at 37 °C for 2 h and then subject to SDS-PAGE analysis on 15% Tris-HCl gel.

### 2.6. Cell-Based DUB Assay

HEK293T cells grown to 70%–80% confluence in six-well plates were transfected plasmids encoding HA-ubiquitin or PLP2-myc or nsp2, nsp2-3 or derivatives either singly or in combination via Lipofectamine PLUS (Invitrogen, CA, USA). At 24 h post-transfection, the cells were treated with MG-132 (Selleck, S2619) at 10 μg/mL for 4 h before collection. The cells were washed by PBS for twice and lysed by RIPA buffer on the shaker at 4 °C for 30 min. The cell lysates were subject to SDS-PAGE and western blot analyses with antibodies to HA or myc epitope. The amount of actin served as a loading control.

### 2.7. Cell-Based Trans- and Cis-Cleavage Assay

For the *trans*-cleavage assay, HEK 293T cells were transfected to express PLP2-myc, or nsp2 D89N, or its derivatives together with the substrate nsp2-3Δ1–399 or nsp2-3Δ1–399 G1166G via Lipofectamine PLUS (Invitrogen, CA, USA). For *cis*-cleavage assay, plasmids coding for nsp2-3 or its derivatives were transfected singly into 293T cells. At 24 h post-transfection, the expression of enzyme PLP2 or nsp2 was subject to western blot analysis by using the cell lysates, whereas the cleavage of substrate was analyzed by immunoprecipitation coupled with western blot. Briefly, the cells were rinsed with cold PBS and lysed with RIPA buffer. The cell debris was removed by centrifugation at 12,000 rpm for 3 min and the supernatants were precleared with the protein A/G agarose (Santa Cruz, CA, USA) on the shaker at 4 °C for 30 min. After that, the cleared supernatants were incubated with 1ug antibodies to FLAG epitope and 12 uL protein A/G agarose (Santa Cruz, CA, USA) at 4 °C overnight. The immunocomplexes were washed for thrice by cold PBS, and the proteins were separated on SDS-PAGE and transferred to PVDF membrane. For western blot analysis, the membrane was blocked by PBST with 5% skimmed milk for 1 h at room temperature and incubated with indicated primary antibody (antibody to HA epitope) at 4 °C overnight. After being washed for thrice by PBST, the membrane was incubated with indicated secondary antibody for 1 h at room temperature. After being washed three times, the membrane was developed with ECL western blot analysis system.

### 2.8. Construction of PRRSV Mutants and Growth Kinetics Analysis

For mutagenesis of the infectious clone of HP-PRRSV strain JXwn06 (EF641008), the mutations were first introduced into shuttle plasmid pEASY-Blunt (Transgen, Beijing, China) contain the fragment A [31] by site-directed QuikChange mutagenesis. After verification by sequencing, digested fragment A was transferred to the infectious clone backbone as described previously [31]. MARC-145 cells grown on six-well plates were transfected with plasmids of either WT or PRRSV mutants. The virus-induced CPE was monitored daily and the cell culture was harvested at 4–5 days post-transfection. The cell lysates were passaged blindly onto fresh monolayers for three passages. The viability of mutants was determined by RT-PCR targeting ORF7 and immunofluorescence to N protein.

The rescued viruses of passage 3 were titrated on MARC-145 cells. For growth kinetics analysis, MARC-145 cells or primary PAMs in six-well plates were infected with PLP2 mutants and parental virus at an MOI of 0.01. For growth on MARC-145 cells, after 1 h of incubation at 37 °C, the cells were first washed with acid buffer (135 mM NaCl, 10 mM KCl, 40 mM citric acid, PH 3.0), followed by once rinse with DMEM. At indicated time points of infection, the whole culture was harvested and freeze-thawed three times to release cell-associated viruses. Samples were then titrated on MARC-145 cells by using the standard endpoint dilution assay according to the Reed-Muench method [44].

### 2.9. Quantitative Real-Time PCR

Primary PAMs in 6-well plates were infected with nsp2 mutants or parental virus at an MOI of 0.1. At 24 h post-infection, total RNAs were extracted from lysates of infected PAMs with TRIZOL reagent (Invitrogen, CA, USA) according to manufacturer’s protocols and dissolved into RNase free water. The concentration of RNA in samples was measured with a Nanodrop lite spectrophotometer (Thermol scientific, USA). 1 μg of total RNAs per sample was used for cDNA synthesis by Fastquant RT Kit (Tiangen, Beijing, China). We conducted qPCR assays using Real-Time SYBR Master Mix Kit (Applied Biosystem, USA) on an AB7500 Real-time PCR system (Applied Biosystem, USA).

### 2.10. Measurement of Secreted TNFα

Primary PAMs in 6-well plates were infected with nsp2 mutants or parental virus at indicated MOI. At indicated time points post-infection, TNF-α in cell culture supernatants was quantified using a commercial sandwich enzyme-linked immunosorbent assay (ELISA) according to the manufacturer’s protocol (Cusabio, Wuhan, China).

### 2.11. Bioinformatics Prediction

The I-TASSER online service tool was used to model the structures of PRRSV strain JXwn06 PLP2 [45]. The PLP2 structures were referred to EAV PLP2 (4IUM) [19], and then its interaction with ubiquitin was analyzed by PyMOL and COOT software.

### 2.12. Quantitative Analysis

The in vitro cleavage efficiency of K48 and K63-linked polyubiquitin chains was expressed as the ratio of the amount of Ub plus Ub2 over total Ub (Ub+Ub2+Ub3+Ub4+Ub5+Ub6+Ub7) by measuring the band density in each lance of SDS-PAGE images. The ratio of Ub2 over Ub1 was calculated by band density of Ub2 over Ub1). The relative DUB activity of PLP2 mutants was measured by the corresponding band density of polyubiquitin conjugates and then normalized against actin and expression level of PLP2. All the data are shown as mean± SEM with 3 independent experiments.

### 2.13. Statistical Analysis

Statistical significance was evaluated by using a two-way analysis of variance (ANOVA). Statistical analyses were performed using GraphPad Prism software (version 5.0). The quantitation of each protein band is measured by Image J software (version 1.5.1).

## 3. Results

### 3.1. The Downstream Flanking Sequence is Critical for the Yield and Solubility of PRRSV PLP2 Protease Domain in E. coli

The N-terminal 215 residues of PRRSV JXwn06 nsp2 was recently reported to exhibit DUB activity when expressed and purified from *E. coli* BL21 cells [41]. This fragment, however, was expressed at a very low level in our hands, preventing further efficient purification. Since the downstream flanking sequence (nsp2 aa. 241–323) is critical for PRRSV nsp2 function during infection [34], we hypothesized that this region might be critical for the folding of PLP2 domain, and if so, a C-terminal extension might improve the solubility and yield of PLP2. Accordingly, we made two additional constructs to include PRRSV strain JXwn06 nsp2 region aa. 12–240 and aa. 12–323 (Figure 1B). These proteins were tagged with a strep II epitope tag at the C-terminus to facilitate purification. When expressed in *E. coli* BL21 cells, PLP2 (12–240)-strep II mostly existed in the pellet, preventing it from efficient affinity purification in large scale (Figure 1B, lane 4). In contrast, PLP2 (12–323)-strepII was well expressed, and a substantial amount was presented in the supernatants (Figure 1B, lane 8). Moreover, this fragment in the sonicated supernatants could be subsequently purified to homogeneity by one-step affinity purification (Figure 1B, lane 10) with a yield of 3–5 mg per 100 mL culture. Thus, we have successfully developed a strategy to realize high-level soluble expression of PRRSV PLP2.

### 3.2. The In Vitro Purified PRRSV PLP2 Can Efficiently Cleave Both K63 and K48-Linked Polyubiquitin Chains Ub3-7 but Displays a Differential Activity in Converting the Respective Ubiquitin Dimers to Monomer

**Figure 2:**
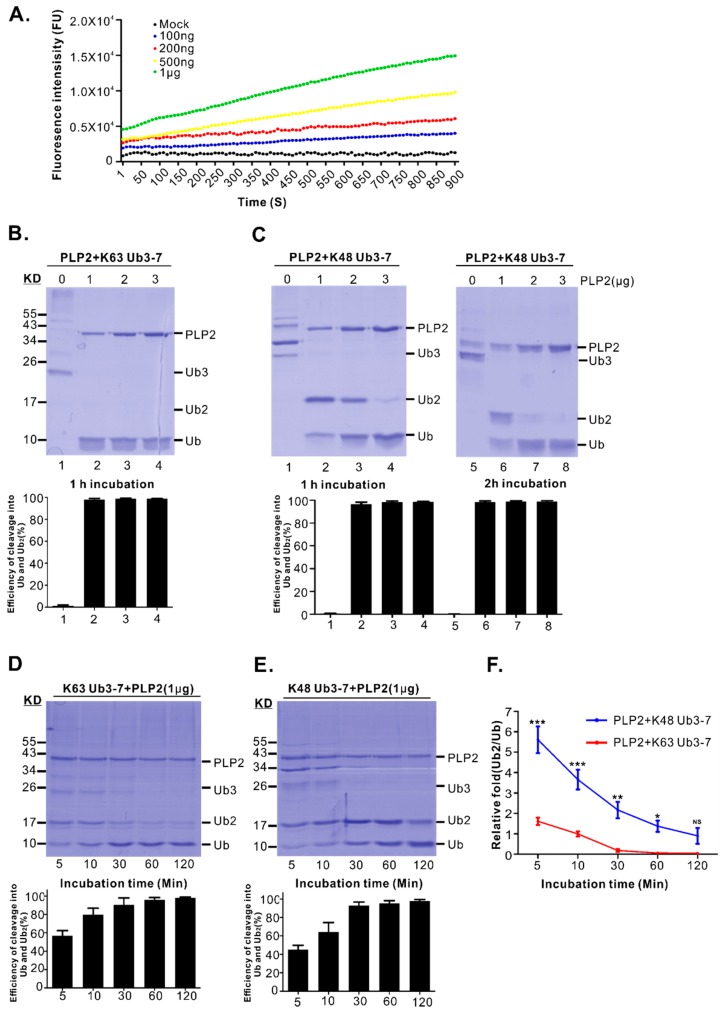
In vitro purified PRRSV PLP2 domain possesses DUB activity. (**A**) In vitro cleavage of 1 μM Ub-AMC with different amounts of PLP2 as indicated in 50 μL reaction buffer at 37 °C. (**B**,**C**) Different amounts of purified PLP2 as indicated was incubated with 2.5 μg K48- or K63-linked Ub3-7 for 1 or 2 h at 37 °C, and the cleavage of the substrate was analyzed by 15% SDS-PAGE gel. The cleavage efficiency was expressed as the ratio of the amount of Ub plus Ub2 over total Ub (Ub+Ub2+Ub3+Ub4+Ub5+Ub6+Ub7) in each lane by measuring the band density. (**D**,**E**) Cleavage kinetics of K48- or K63-linked Ub3-7 in a reaction containing 1 μg PLP2, 2.5 μg K48- or K63-linked Ub3-7 at 37 °C. The cleavage of Ub3-7 was analyzed by 15% SDS-PAGE gel. The quantitation was measured the same as in B and C, and the results were shown under the corresponding figures. (**F**) The ratio of Ub dimers over monomer in D and E was quantified by the band density. All the data were presented as means ± standard error of means (SEM) of three independent experiments. (*, *p* < 0.05, **, *p* < 0.01, ***, *p* < 0.001, NS, no significant difference).

The DUB activity of purified PLP2 was subsequently investigated in a series of in vitro assays. We first tested its ability to hydrolyze Ub-AMC, an ubiquitin conjugated aminomethylcoumarin fluorophore (AMC). By monitoring the release of AMC, the DUB activity was measured. As shown in Figure 2A, the purified PLP2 exhibited DUB property in vitro with better activity achieved at a higher concentration (1μg). Next, we examined the cleavage of a specific type of polyubiquitin chains (Figure 2B,C). Overall, when the total amount of processed Ub monomer and dimers (Ub+Ub2) was calculated, PRRSV PLP2 exhibited similar efficiency in cleaving both K48- and K63-linked polyubiquitin chains Ub3-7 (% cleavage efficiency = Ub1+Ub2/Ub1-7) (Figure 2B,C). However, PRRSV PLP2 display a differential activity converting K48 and K63 linked ubiquitin dimers into monomers. The results showed that the cleavage into monomer of K48-linked Ub3-7 was relatively inefficient (Figure 2C) with the reaction taking place in a dose and time-dependent manner (Figure 2C). For example, at a lower dose of PLP2 (1 μg), K48-linked Ub3-7 was mainly cleaved into Ub dimers (Figure 2C, lane 2), even if the incubation time was extended to two hours (Figure 2C, lane 6). However, when the dose increased (3 μg), PLP2 could hydrolyze K48 Ub3-7 efficiently into monomers within 1 h (Figure 2C, lane 4). The difference in kinetics toward cleaving K63 versus K48 Ub3-7 was further investigated in a more detailed time-dependent manner from 5 to 120 min at a concentration of 1 μg for PLP2. The result showed that PLP2 was able to quickly cleave both K63 and K48 Ub3-7 within minutes (Figure 2D,E), but the conversion from ubiquitin dimer to monomer was several fold faster for PLP2 K63 DUB activity (Figure 2D–F).

### 3.3. Identification of Residues Critical for the PLP2 DUB Activity

**Figure 3:**
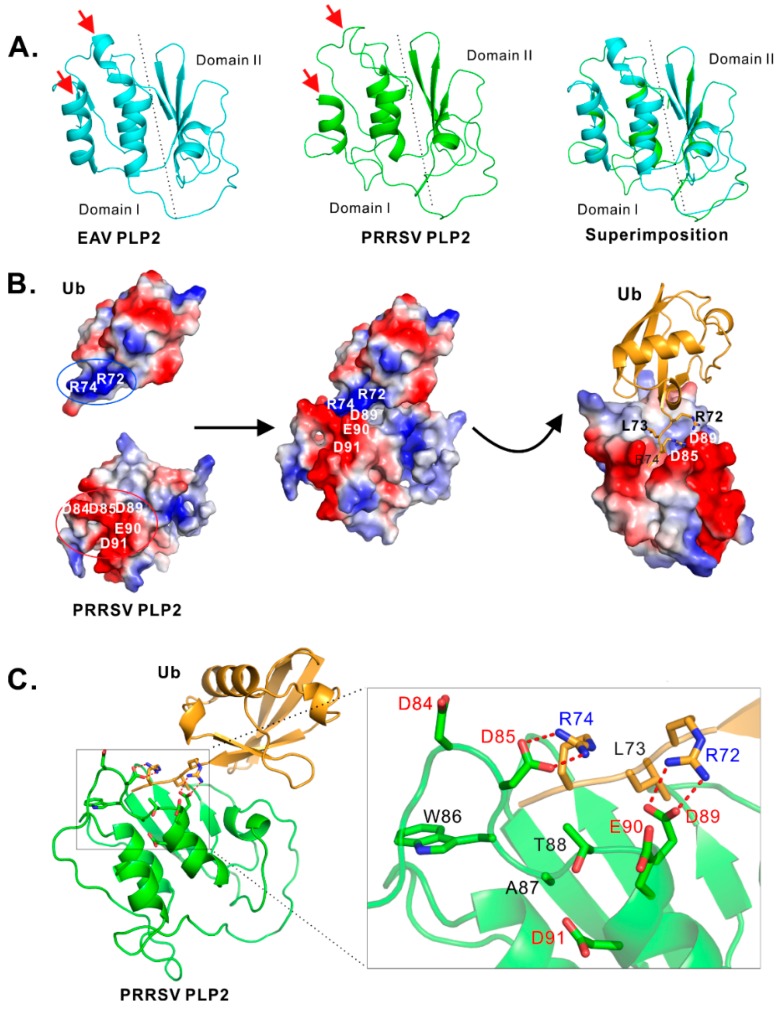
Bioinformatics analysis of the potential interaction sites between PLP2 and Ub. (**A**) Predicted structure of PRRSV PLP2, and structure superimposition with EAV PLP2 (PDB ID: 4IUM). The arrows indicate a structure difference. (**B**). Cartoon form of both Ub and PRRSV PLP2 with the potential contact area indicated. (**C**) Prediction of the potential contact sites with the dots showing the hydrogen bond.

To identify critical residues that are potentially important for PLP2 DUB activity, we modeled the structure of PLP2 core domain with the online program I-TASSER [45] by using EAV PLP2 (PDB ID: 4IUM) as a model. The homology remodeling revealed that PRRSV strain JXwn06 PLP2 (aa. 1–152) has an overall similar structure to that of the counterpart EAV PLP2, except for two minor structural differences as indicated by the red arrows (Figure 3A).

The bioinformatics analysis also identified an acidic cluster (D84DWATDED91) that has potential contact with the positively charged C-terminus of ubiquitin (Figure 3B). In particular, the Ub residues R72 and R74 form a positively charged surface, whereas PRRSV PLP2 residue D84, D85, D89, E90 and D91 form a negatively charged region. The modeling showed that the charged C-terminus is inserted into a grove formed by negatively charged residues D85, E90, and D89 of PLP2 (Figure 3B). Further, a closer look into the contact sites revealed that both PLP2 D85 and D89 can potentially interact with R74 and R72 respectively through potential hydrogen bond and electrostatic interactions (Figure 3C). In contrast, D84 and D91 are in a position that is far away from Ub C-terminus (Figure 3C).

**Figure 4:**
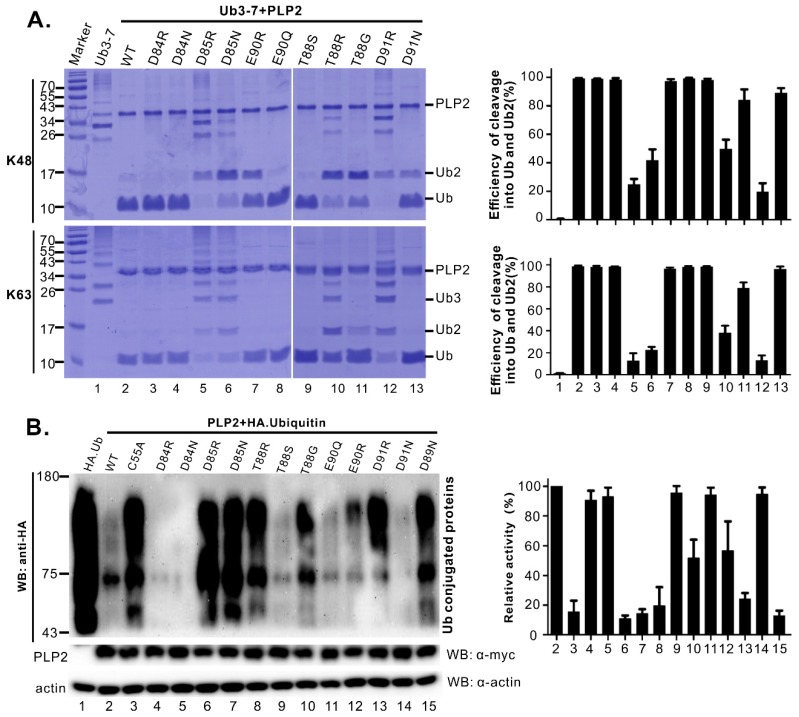
Mutational analysis of acidic cluster on PLP2 DUB activity. (**A**) 3 μg in vitro purified recombinant wild-type PLP2 or the mutants were incubated with 2.5 μg K48- and K63-linked polyubiquitin chains in the reaction buffer for 2 h at 37 °C and the cleavage efficiency was examined in a 15% SDS-PAGE gel. The cleavage efficiency was measured by the total amount of Ub plus Ub2 over total Ub (Ub+Ub2+Ub3+Ub4+Ub5+Ub6+Ub7) in each lane according to the band density. (**B**) Plasmid coding for HA-ubiquitin was either singly transfected or co-transfected with plasmids encoding wild-type PLP2-Myc or PLP2-Myc mutants into 293FT cells. At 24 h post-co-transfection, the cell lysates were delivered to standard western blot analysis with antibodies to either HA, myc, or β-actin. The figure on the right shows the quantification analysis of DUB activity that was normalized against actin and expression level of PLP2. The data were presented as means ± standard error of means (SEM) of 3 independent experiments.

For this identified acidic cluster, the residues D84, D85, W86, A87, D89, and D91 are highly conserved among various PRRSV strains, whereas the residues T88 and E90 are highly conserved among type II PRRSV strains but are replaced with the respective residues S and Y in type I PRRSV strains (Figure S1) [34]. Among these residues, both W86 and D89 have been shown to be critical for the *trans*-cleavage of nsp2/3 [34]. Thus, we, therefore, selected other 5 residues (D84, D85, T88, E90, D91) for further mutational analysis with a purpose to distinguish DUB from its *trans*-cleavage activity. The acidic residues were changed to either the residue asparagine (N) or to the opposite charge residue arginine (R), whereas T88 was replaced with either serine (S), glycine (G), or arginine (R). The corresponding mutants were expressed and purified from *E. coli* BL21 cells, and their enzymatic activities were tested by the in vitro DUB assay (Figure 4A) and the quantified relative activity was shown at the right of corresponding figures.

The results showed that the mutations of the residues D84 and E90 (e.g., D84N, D84R, E90R, E90Q, etc.) did not have much effect on PLP2 DUB activity, as the corresponding mutants could efficiently cleave K63 or K48 polyubiquitin chains into monomers (Figure 4A, lanes 3, 4, 7, and 8). In contrast, mutation of the residue D85 to either N or R largely blocked the cleavage of Ub3-7 with only a very small portion cleaved into Ub dimer or monomer (Figure 4A, lane 5 and 6). The mutational effect of D91 was amino acid-dependent. The D91R mutation largely blocked the PLP2 DUB activity (Figure 4A, lane 12), whereas the mutation D91N did not affect the cleavage much (Figure 4A, lane 13). For the residue T88, substitution with R (T88R) largely ablated the DUB activity (Figure 4A, lane 10), whereas the mutation to serine (T88S) did not have an effect (Figure 4A, lane 9). The T88G mutation did not have a large effect on the ability of PLP2 to process the K63 polyubiquitin chains (Figure 4A, lane 11, bottom panel), but it partially crippled the ability of PLP2 to cleave the ubiquitin dimer (Figure 4A, lane 11, top panel), suggesting a differential sensitivity.

We next used a cell-based assay to further check on the mutational effect on PLP2 DUB activity within mammalian cells (Figure 4B). At the same time, the mutants PLP2 C55A and D89N (Figure 4B, lanes 3 and 15), which have been shown to be defective of *trans*-cleavage activity [34], were used as negative control. The HA-tagged ubiquitin was transiently expressed in HEK 293T cells together with PLP2 or PLP2-derived mutants. At 24 h post-transfection, the cell lysates were collected to assess the level of ubiquitination by western blot analysis with antibodies to HA epitope (Figure 4B). The results showed that the mutants D84R, D84N, T88S, E90Q, and D91N were DUB active (Figure 4B). In contrast, the mutants D85R, D85N, T88R, and D91R lost the DUB activity, together with the control mutants PLP2 C55A and D89N (Figure 4B). On the other hand, the mutations E90R and T88G partially crippled the PLP2 DUB activity (Figure 4B, lanes 11 and 9).

Overall, the cell-based results are largely consistent with the in vitro DUB assay. In addition, the results of mutagenesis studies are in accordance with the bioinformatics prediction, except for several mutations (e.g., T88R, E90R, and D91R), which will be discussed in the discussion section.

### 3.4. Differentiation of DUB Activity from Trans- and Cis-Cleavage Activity

**Figure 5:**
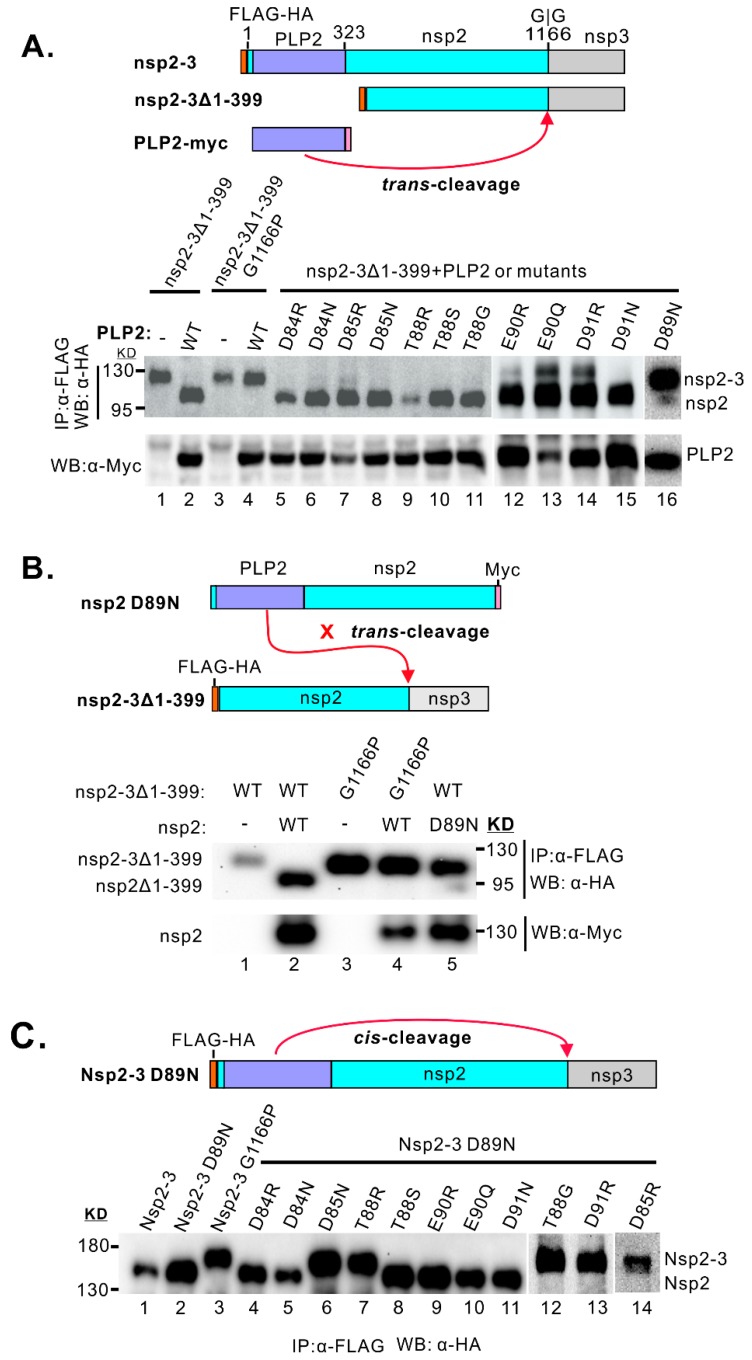
Effect of point mutations on PLP2 protease activity. (**A**) *Trans*-cleavage assay. Schematic diagram of constructs was shown on the top. WT PLP2 or its derivatives were co-expressed with the substrate nsp2-3Δ1–399 in 293T cells by transfection. The mutant nsp2/3Δ1–399 G1166P served as non-cleavage control. The cleavage of nsp2-3Δ1–399 was examined by western blot with antibodies to HA after immunoprecipitation with anti-FLAG monoclonal antibodies. The PLP2 was analyzed by western blot with anti-c-myc antibodies. (**B**) *Trans*-cleavage activity of the mutant nsp2 D89N. The analysis was the same as in (**A**). (**C**) *Cis*-cleavage assay of nsp2 mutants. Nsp2-3 D89N or its derivatives were expressed in 293T cells by transfection, and the processing of nsp2 was analyzed by western blot with antibodies to HA after immunoprecipitation with anti-FLAG monoclonal antibodies.

We next investigated whether the same mutation affects the PLP2 *cis*- or *trans*-cleavage activity, which can be important for the processing of PRRSV polyprotein nsp2-3 during infection. To address this question, we performed the cell-based assay by co-expressing PLP2 or its derivatives with the substrate nsp2-3Δ1–399 lacking the PLP2 domain in transfected 293FT cells (Figure 5A). For a negative substrate control, the PLP2 cleavage site at the nsp2-3 junction was mutated to make the construct nsp2-3Δ1–399 G1166P (Figure 5A). As shown in Figure 5A, except for the control D89N, all other PLP2 mutants were capable of efficiently processing the nsp2-3 polyprotein, suggesting that the mutations did not affect the *trans*-cleavage activity of PLP2. Thus, the mutations (e.g., D85N, D85R, E90R, D91R, T88R, etc.) that affected the DUB activity can be used to differentiate the DUB activity from *trans*-cleavage activity.

We next investigated whether the same mutations affect the PLP2 *cis*-cleavage activity. Since *cis*-cleavage is essentially an intramolecular interaction, the mutational effect was evaluated by using the full-length nsp2-3 polyprotein precursor as reported previously [34]. In addition, since both *cis*- and *trans*-cleavage activities can direct the nsp2-3 cleavage [34], the *trans*-cleavage has to be silenced in the first place before proceeding to test the effect on *cis*-activity. Here, we took advantage of the D89N mutation, which is able to decouple the nsp2 *cis*- from *trans*-cleavage activity by using PRRSV strain VR-2332 [34]. Consistently, the D89N mutation strongly reduced the *trans*-cleavage activity of PLP2 to process nsp2-3Δ1–399 in *trans* in the cotransfection assay (Figure 5A, lane 16), and the cleavage efficiency was only about 2–3%. To make sure the full-length nsp2 carrying D89N mutation also behaves the same as seen for the PLP2 fragment (Figure 5A, lane 16), we introduced this mutation into the full-length nsp2 of PRRSV strain JXwn06. As expected, the full-length nsp2 carrying D89N mutation also failed to process in trans the polyprotein nsp2-3 lacking the protease domain (nsp2-3Δ1–399) in the co-transfected cells (Figure 5B, lane 5), suggesting the *trans*-cleavage activity is blocked. When the same mutation was introduced into the nsp2-3 precursor (nsp2-3 D89N), it, however, did not affect the processing in cis of nsp2 from nsp2-3 D89N (Figure 5C, lane 2). Thus, this result is in agreement with the previous report based on PRRSV strain VR-2332 [34]. Next, we introduced the corresponding acidic cluster point mutations into the background nsp2-3 D89N (Figure 5C), which allows only cis-processing of nsp2. To our surprise, the mutations (e.g., D85N, D85R, T88R, and D91R) that largely ablated the DUB activity also blocked the cis-activity of PLP2 (Figure 5C, lanes 6, 7, 13, and 14, respectively). In contrast, neither D84N nor D84R affected the cis-cleavage activity (Figure 5C, lane 4 and 5). Notably, the mutation T88G could selectively block the PLP2 cis-cleavage activity (Figure 5C, lane 12), as the mutant PLP2 T88G was still equipped with trans-cleavage activity (Figure 5A, lane 11) and largely DUB activity (Figure 4A, lane 11). Together, these results suggest that the requirement for PLP2 DUB activity somehow is more closely related to that for cis-cleavage activity, rather than the trans-cleavage activity. In addition, T88G is a critical mutation that can differentiate the cis-cleavage activity from DUB and trans-cleavage activity.

### 3.5. The PLP2 Cis-Cleavage Activity is Dispensable for HP-PRRSV Strain JXwn06 Viability in Cell Culture

The mutational effect on viral replication was tested by introducing the corresponding point mutations into the infectious cDNA clone of HP-PRRSV strain JXwn06 in a DNA-launched system. The recombinant plasmids were transfected into MARC-145 cells for virus recovery. Our analyses by reverse genetics of PLP2 mutants led to the findings as follows (Table 1). (i) Mutations (D85N, D85R, T88R, and D91R) that largely blocked the DUB activity in cell-based assay were all lethal to the virus, (ii) mutations (D84R, D84N, T88S, and E90Q) that did not have an effect on DUB activity did not affect viral viability, (iii) the point mutation T88G that selectively blocked the PLP2 *cis*-activity did not affect viral viability in cell culture, and (iv) the mutation D91N and E90R, which did not affect either of the PLP2 activities, somehow did not allow the rescue of viable virus. Together, our results strongly suggest a dispensable role of the PLP2 *cis*-activity for PRRSV viability. On the other hand, the fact that the *cis*-cleavage activity is not required for PRRSV replication suggests that the lethal phenotype of several point mutations (D85N, D85R, T88R, and D91R), which blocked both DUB and *cis*-activity, is not due to a block in *cis*-cleavage activity, but may be linked to the inactivation of PLP2 DUB activity.

### 3.6. HP-PRRSV-Induced Production of TNF-α and IL-1β is Strongly Associated with nsp2 that is Independent of PLP2 DUB Activity

**Figure 6:**
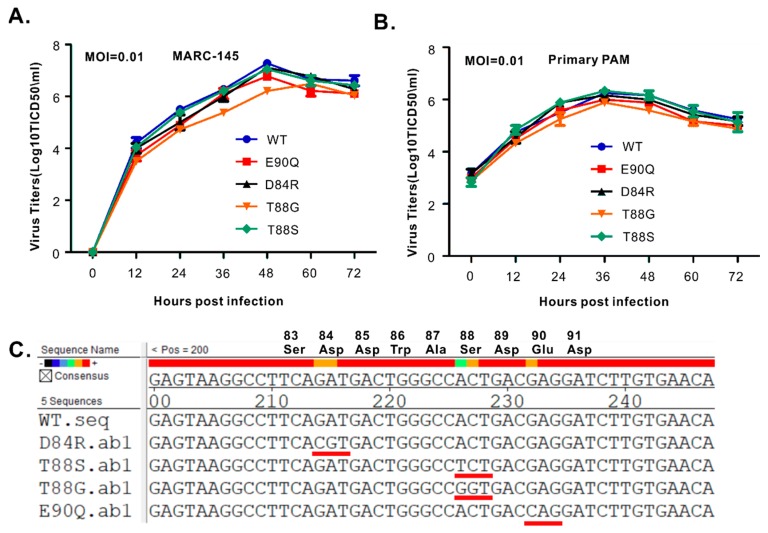
The PLP2 *cis*-activity is not required for PRRSV replication in cell culture. (**A**,**B**) MARC-145 cells or PAMs were infected with indicated viruses at an MOI of 0.01. Virus titers at different time points, as indicated, were determined by endpoint dilution assay. The error bars indicate standard error of the mean (SEM). (**C**) Sequencing result of the PLP2 region with the sequences aligned with DNAstar. The underlined codons represent the introduced mutations during mutagenesis.

**Figure 7:**
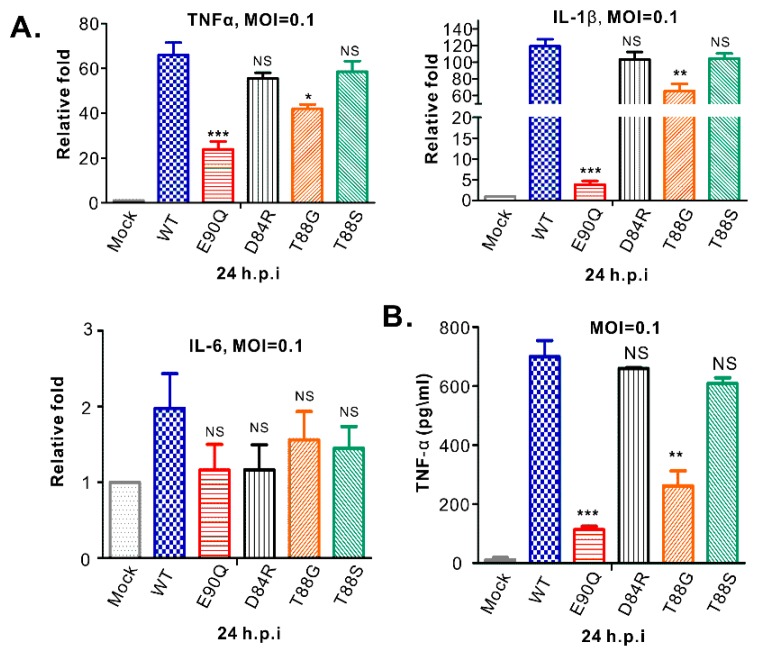
Effect of PLP2 mutations on pro-inflammatory cytokine expression in PRRSV-infected PAMs. PAMs were either mock-infected with DMEM or infected with indicated viruses. The cells were harvested at 24 hpi, and the expression of indicated cytokine mRNAs was determined by using relative qPCR and normalized against the expression level of endogenous PPIA (**A**). The protein level of TNF-α in the infected supernatants was measured by ELISA (**B**). Data are shown as the mean ± SEM of there independent experiments. (*, *p* < 0.05, **, *p* < 0.01, ***, *p* < 0.001, NS, no significant difference).

Four viable mutants of passage 3 (P3) were chosen for growth kinetics analysis in both MARC-145 cells (Figure 6A) and primary PAMs (Figure 6B). All the mutants tested showed relatively similar growth properties to the parental virus, except for the mutant T88G, which exhibited reduced growth in MARC-145 cells by about half a log and a slight decrease in PAMs although being statistically insignificant (Figure 6B). To test the stability, we sequenced the PLP2 mutation region at the end of experiments or P4 viruses. The results showed that the mutations were stable (Figure 6C). In addition, we did not see any other compensating mutations arising within nsp2. Since PRRSV nsp2 is involved in antagonizing host innate immunity and the PLP2 mutants did not have an apparent growth defect in primary PAMs, we tested whether mutations have an effect on inflammatory cytokine production rather than on interferon signaling. In particular, we focused on the expression and secretion of TNF-α, IL-1β, and IL-6 (Figure 7), the most potent inflammatory cytokines. To this end, primary PAMs were infected with WT or mutant viruses with an MOI of 0.1. At 24 h post-infection, the mRNA level of TNF-α, IL-1β, and IL-6 were measured by relative quantitative RT-PCR normalized against peptidylprolyl isomerase A (PPIA), a molecule that has been previously used for internal control [46,47,48]. The results showed that PRRSV strain JXwn06 significantly upregulated the mRNA expression of both TNF-α and IL-1β (Figure 7A). On the other hand, the effect on IL-6 was not impressive (Figure 7A). Interestingly, the mutant E90Q exhibited greatly reduced ability to induce expression of both TNF-α and IL-1β, and to a lesser extent by the mutant T88G (Figure 7A). Similarly, none of the mutants affected the level of IL-6 (Figure 7A). We also measured the secretion of these cytokines in the infected supernatants by ELISA. Consistent with a reduction at the mRNA level, we observed a significant drop of TNF-α in the cell culture supernatant from primary PAMs infected with the mutants T88G and E90Q (Figure 7B). For IL-1β and IL-6, we tried several commercial kits, and unfortunately, they did not work well in our hands.

**Figure 8:**
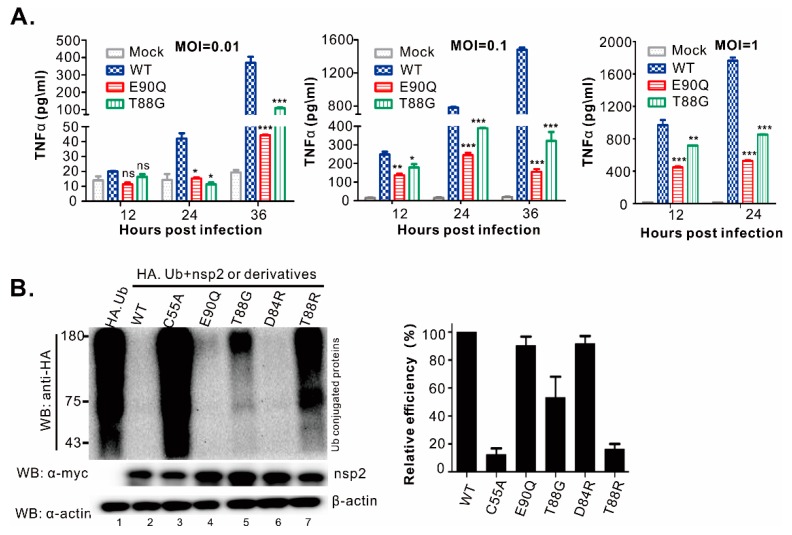
HP-PRRSV-induced TNFα secretion is strongly associated with nsp2 but independent of its DUB activity. (**A**) PAMs were either mock-infected by culture medium or infected with indicated viruses at different MOI. Cell supernatants were harvested at indicated time points post-infection, and the levels of TNF-α were analyzed by ELISA. Data are shown as mean ± SEM. (*, *p* < 0.05, **, *p* < 0.01, ***, *p* < 0.001, ns, no significant difference). (**B**) 293T cells were transfected to express HA-ubiquitin or in combination with full-length nsp2 or its derivatives. At 24 h post-co-transfection, the cell lysates were delivered to standard western blot analysis with anti-HA, c-myc or β-actin antibodies. The histogram shows the quantitation of each lane after being normalized against β-actin and the expression level of nsp2. The data are shown as mean ± SEM (*n* = 3) of 3 independent experiments.

To test whether the effect was contingent on the infection dose, we chose E90Q and T88G for further analysis (Figure 8). The results showed that either MOI of 0.01, 0.1, or 1 could significantly reduce the secretion of TNF-α in the infected supernatants (Figure 8A). When compared with WT, this reduction is by more than 70–80% for the mutants E90Q and T88G at 24 h post-infection or later, Moreover, the E90Q mutant had a greater ability to reduce the production of TNF-α (Figure 8A).

Finally, to further evaluate whether the observed effect on pro-inflammatory cytokine production is associated with altered PLP2 DUB activity, we introduced the corresponding mutations (E90Q, T88G, D84R, and T88R) into the full-length nsp2 rather than merely into the PLP2 fragment (Figure 8B). The deubiquitinating ability of nsp2 or its derivatives was then tested in transfected 293T cells by co-expression with HA-ubiquitin. Consistent with the phenotype exhibited by the truncated nsp2, the mutant nsp2 E90Q (Figure 8B, lane 4) behaved just like WT nsp2 (Figure 8B, lane 3), and nsp2 T88G was largely DUB-active (Figure 8B, lane 5). In contrast, the catalytic site mutation C55A blocked the DUB activity of the full-length nsp2 (Figure 8B, lane 3). Together, the above data provided strong evidence on a critical role of PRRSV nsp2 in inducing TNF-α and IL-1β during infection, and also this property of nsp2 is independent of the PLP2 DUB activity.

## 4. Discussion

In this report, we developed a strategy to obtain high-level soluble expression of PRRSV strain JXwn06 PLP2 in *E. coli* BL21 cells for assaying its DUB activity in vitro. The in vitro and cell-based assays uncovered important biochemical properties of PLP2, including efficient cleavage ability towards both K48- linked and K63- linked polyubiquitin chains and a surprisingly shared requirement for both *cis*-cleavage and DUB activity. Moreover, we identified mutations that could distinguish the three activities of PLP2. Further reverse genetics analysis revealed dispensability of *cis*-activity for PRRSV replication. In contrast, viruses carrying point mutations that largely blocked the DUB activity were not viable, pointing to a potentially critical role of PLP2 DUB in PRRSV replication. During characterizing the viral mutants, we also unexpectedly discovered a DUB-independent regulation of pro-inflammatory cytokine production by PRRSV nsp2. Together, these findings further deepen our understanding of the biological properties and function of PRRSV PLP2 and nsp2 in virus life cycle and have implications in understanding viral pathogenesis and in vaccine development. The relevant significance or insights are discussed below.

### 4.1. Insight into the Biochemical Properties of PRRSV PLP2

It is now known that PRRSV PLP2 possesses at least *cis*-, *trans*-cleavage, and DUB activities and that the *trans*-activity requires the nsp2 region aa. 47–240 [34]. In this report, we went much further to reveal several important biological aspects of this particular protease. Beginning with the in vitro purification, we found that the downstream flanking sequence (nsp2 aa. 240–323) was critical for the yield and solubility of PLP2 domain in the prokaryotic expression system (Figure 1B). Although a previous study reported that the PRRSV strain JXwn06 PLP2 (nsp2 aa. 1–215) is sufficient for DUB activity in vitro, but it did not provide description about the expression level and purification of this fragment [41]. In our hand, this fragment was unfortunately poorly expressed in *E. coli* BL21 cells despite attempts for optimization of expression conditions. Although truncation is usually the way for protein expression, we used an unconventional approach to solve the problem, making a C-terminal extension dramatically improved the PLP2 yield and solubility (Figure 1B). Our results suggest that the flanking sequence likely plays an important role in the folding of PLP2 core domain, and this is consistent with our previous finding that deletion of nsp2 aa. 240–323 is lethal to PRRSV strain VR2332. The realization of high-level soluble expression also paves the way for future structural studies and in vitro anti-PLP2 drug screening.

Previous studies have shown that PRRSV PLP2 possesses differential DUB activity toward cleaving K48 and K63 ubiquitin dimers into monomer. Our results are in general in agreement with this finding [41]. Different from the previous study, we found that PRRSV PLP2 is able to cleave ubiquitin dimer but just in a dose and time-dependent manner. This difference is likely attributed to the PLP2 flanking sequence (aa. 240–323) (Figure 2), which can promote the folding of PLP2 core domain, leading to increased cleavage efficiency toward K48 polyubiquitin chains. In addition, we found that PRRSV strain JXwn06 PLP2 can equally efficiently cleave K48 and K63-linkd Ub3-7 into dimers and monomer.

We also identified critical residues for PRRSV PLP2 DUB activity. D85, an amino acid that is highly conserved among PRRSV strains [34], was revealed to be a key residue for PRRSV PLP2 DUB activity (Figure 4). Either a conserved (D85N) or non-conserved (D85R) substitution led to a significant destruction of PLP2 DUB activity (Figure 4). The mutational effect also appears specific, as similar mutations of the nearby residue D84 (D84N and D84R) that is also highly conserved did not have an effect (Figure 4). This result is also in accordance with the bioinformatics prediction (Figure 3). The mutational effect of other residues is contingent on the amino acids used for substitution. For example, the mutations D91R and T88R largely blocked the PLP2 activity and E90R partially blocked the DUB activity (Figure 4). We suspect that the mutational effect of D91R is most likely indirect and could result from electrostatic interaction with E90 to change the local structure. In a similar manner, the mutation T88R can potentially interact locally with E90, D91, or D85, leading to local conformational change of PLP2, which may have an adverse effect on PLP2 function. This also partially explains why T88S, E90Q, and D91E did not have an effect. Most interestingly, we found that the T88G mutation can selectively block the *cis*-cleavage activity (Figure 5C), without affecting the *trans*-cleavage activity (Figure 5A) and only partially blocking the DUB activity in either truncated (Figure 4B) or full-length form (Figure 8B). This is the first time to report a point mutation that distinguishes *cis*-cleavage from other activities and paves the way to test the importance of *cis*-cleavage activity in PRRSV infection.

It has been viewed that hydrolysis of ubiquitin substrates takes advantage of the *trans*-activity of viral DUBs. Surprisingly, we observed an intriguing relationship between PRRSV PLP2 DUB and *cis*-activity. That is, several mutations that largely blocked the PLP2 DUB activity were found to also ablate its *cis*-activity, and these include the mutants D85N, D85R, T88R, and D91R (Figure 4 and Figure 5C). In contrast, none of these mutations had an effect on the PLP2 *trans*-activity cleaving nsp2-3 polyprotein (Figure 5A). Thus, it seems that the requirement for PRRSV PLP2 DUB activity is somehow more closely related to *cis*-activity, suggesting that they may share some intrinsic common mechanistic details in terms of recognition and cleavage of the substrates. To our knowledge, this is the first report revealing such intimate relationship between DUB and *cis*-activity concerning OTU cysteine proteases. The molecular basis for these observations is currently not clear, but hopefully, elucidation of the three-dimensional structure of PRRSV PLP2 may help resolve the puzzle in the future.

### 4.2. The Role of Cis-Cleavage and DUB Activities of PLP2 in PRRSV Infection

A long-asked question is whether the PLP2 DUB and *cis*-cleavage activities are essential for PRRSV infection. The inability to differentiate the enzymatic activities by point mutations has limited the evaluation of the contribution of individual enzymatic activities. Despite the reports of several studies on the PLP2 DUB activities [20,21,41,42], none of them established a strong correlation between DUB and viral replication, as they did not rule out the mutational effect on both *trans*- and *cis*-cleavage activities. By taking advantage of site-directed mutagenesis, we in the first place provided strong evidence to suggest that the PLP2 *cis*-activity is not necessary for viral replication (Figure 6). This is evidenced by the specific point mutation T88G, which selectively blocked the PLP2 *cis*-cleavage activity but allowed the successful rescue of viable virus. Moreover, this mutant exhibited similar growth rate to WT in primary PAMs (Figure 6). In contrast, the mutations (e.g., D85N, D85R, T88R, D91R, etc.) that largely disarmed the PLP2 DUB activity were lethal to PRRSV in MARC-145 cells. Although these mutations also crippled the *cis*-cleavage activity of PLP2 at the same time, the successful rescue of T88G mutant strongly argues against an essential contribution of inactive *cis*-activity to the non-viability phenotype. In addition, the lethal phenotype is less likely due to a potential defect of deISGylation activity of PLP2, as it has been reported that the HP-PRRSV strain JXwn06 PLP2 used in this study has very limited deISGylation activity [41] and that ISGylation was not observed in PRRSV-infected MARC-145 cells [21], a cell line that was used for virus rescue. Thus, there appears to exist a strong correlation between the lethal phenotype of these mutations and the essential role of PLP2 DUB activity in PRRSV infection.

Sun et al. recently characterized the nsp2 OTU domain of a European PRRSV strain SD011-08 (type I strain) and reported that PLP2 inhibits NF-κB activation through its DUB activity [20]. They also mutated the residues in the acidic cluster, including the residues D458, D459, S462, D463, D465, etc., corresponding to the residues D84, D85, T88G and D91 in this study, respectively [20]. Consistent with our studies, the mutations D459A and D463A were lethal to PRRSV, whereas D458A, S462A, and D465A allowed the successful rescue of the viable virus [20]. In these studies, the mutant D458A did not affect the viral growth, whereas S462A and D465A crippled the virus in MARC-145 cells [20]. Sun et al. further correlated the growth property with the ability to inhibit NF-κB activation and found that the mutations that were lethal to the virus were these capable of completely inhibiting NF-κB activation, whereas the mutations S462A and D465A unable to inhibit the activation as WT lead to viable virus [20]. However, they did not further test the mutational effect of these mutants on *cis*- and *trans*-cleavage activity and the DUB activity. Our studies here suggest that the lethal phenotype is not linked to an impairment of *cis*- or *trans*-cleavage activity, but rather related to an effect on PLP2 DUB activity. However, because these are negative results, we could not rule out the possibility that the DUB-inactivating mutations may have other unexpected consequences on nsp2, or both, which renders the virus non-viable. On the other hand, the mutations E90R and D91N, which did not affect much the PLP2 DUB activity, did not allow the rescue of viable viruses. This result suggests that the mutations may have an adverse effect on other functions of nsp2, highlighting the complexity of mutational effect on the function of PLP2 or nsp2. Another interesting observation is that the partial ablation (T88G) of PLP2 DUB activity did not affect viral viability whereas a near-complete block (e.g., D85N, D85R, T88R, D91R, etc.) was lethal to the virus. These results may suggest that there exists a threshold of DUB activity required for PRRSV replication, in which the DUB activity may be required to remove polyubiquitins from substrates. Future study will be directed to investigate detailed mechanisms by which the PRRSV PLP2 plays during infection.

### 4.3. Insight into the Induction of Pro-Inflammatory Cytokine Production During PRRSV Infection

Infections by PRRSV often cause de-regulation of inflammatory cytokine production [49]. For the Chinese HP-PRRSV, it inhibits inflammation in early infection, but induces inflammatory storm during the late stage in pigs, contributing to lung injury and interstitial pneumonia [49,50]. The regulatory mechanisms of this process have remained poorly understood, but it is known that several nonstructural proteins, including nsp1α, nsp1β, nsp4, and nsp11, are capable of inhibiting inflammation response [51,52,53,54,55,56,57,58], whereas the structural proteins N and E promote inflammation [59,60]. PRRSV nsp2 is a type I interferon signaling antagonist [20] and recently implicated in the modulation of pro-inflammatory cytokine production during infection [20,35,36]. Liu et al. [36] reported that mutants of HP-PRRSV strain BB carrying deletion of nsp2 region aa. 323–433 or aa. 628–747 had the reduced ability to induce the expression of inflammatory cytokines IL-1β, TNF-α, and IL-6 in PAMs. However, the corresponding mutants also exhibited reduced growth titer by 0.5 to 1 log when compared with the parental virus. Moreover, down-regulation is quite subtle (<30%). On the other hand, the single-site substitution represents a nice approach by which it can minimize the risk of gross conformational alteration as a result of mutagenesis. At the beginning of this study, we did not mean to study the role of nsp2 in inflammatory cytokine induction, but with two independent point-mutation mutants (T88G and E90Q) at hand, we unexpectedly established a strong link of nsp2 to PRRSV-induced induction of pro-inflammatory cytokines (Figure 7 and Figure 8). The mutants T88G and E90Q retained the similar growth rate to the parental virus JXwn06 in primary PAMs, but exhibited significantly reduced ability to induce TNF-α and IL-1β at both mRNA and protein levels (Figure 7 and Figure 8). Moreover, this reduction rate was by more than 70% at 24 hpi or later (Figure 8A), suggesting a decisive role of nsp2 in modulating the production of TNF-α and IL-1β during infection. Different from the previous study [36], we did not observe a change in expression level of IL-6.

The crippled ability to induce TNF-α and IL-1β cannot be attributed to an impairment of PLP2 DUB activity, as the mutant nsp2 E90Q was fully DUB active (Figure 8B). In addition, because viral DUBs often negatively regulate host innate immunity, it is expected that inactivation of DUB activity should promote the inflammatory cytokine production as reported by several cases [12,15,16,19,61]. Our results, however, showed the opposite, the mutant T88G with partially crippled DUB activity showed reduced production of TNF-α and IL-1β (Figure 7 and Figure 8). Thus, with two independent nsp2 mutants (T88G and E90Q) showing the similar phenotype, our results provide strong evidence to suggest a DUB-independent regulatory mechanism by PRRSV nsp2 in inducing TNF-α and IL-1β production. Together, our findings further deepen our understanding of the biological properties and function of PRRSV nsp2 and also provide clues into the pathogenic mechanisms of HP-PRRSV. Additionally, the results have implications in vaccine development by modifying nsp2 to reduce cytokine-induced lung injury. In the near future, it will be interesting to address molecular mechanisms of how the point mutations in nsp2 lead to crippled ability of PRRSV to induce pro-inflammatory cytokines.

## Figures and Tables

**Table 1:** The association between various PLP2 activity and virus viability.

PLP2 Mutants	DUB Activity	*Cis*-Cleavage	*Trans*-Cleavage	Viral Viability	Cytokine Production
D84R	Active	√	√	viable	No effect
D84N	Active	√	√	viable	ND
D85R	Largely blocked	-	√	nonviable	
D85N	Largely blocked	-	√	nonviable	
T88R	Largely blocked	-	√	nonviable	
T88S	Active	√	√	viable	No effect
T88G	Partially blocked	-	√	viable	TNF-α, IL-1β 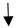
E90R	Slightly blocked	√	√	nonviable	
E90Q	Active	√	√	viable	TNF-α,IL-1β 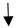
D91R	Largely blocked	-	√	nonviable	
D91N	Active	√	√	nonviable	

√: active, -: blocked, ND: not determined, 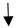: downregulation.

## Supplementary Materials

The following are available online at https://www.mdpi.com/1999-4915/11/10/896/s1, Figure S1: The sequence alignment of the PLP2 acidic cluster title.
