# Supplementary material for: Characterizing the PRRSV nsp2 Deubiquitinase Reveals Dispensability of Cis-Activity for Replication and a Link of nsp2 to Inflammation Induction
Source: Viruses. 2019 Sep 26;11(10):896. doi: 10.3390/v11100896 (PMC6832237; doi:10.3390/v11100896)
Supplement: Supplementary file 1 [file viruses-11-00896-s001.pdf]

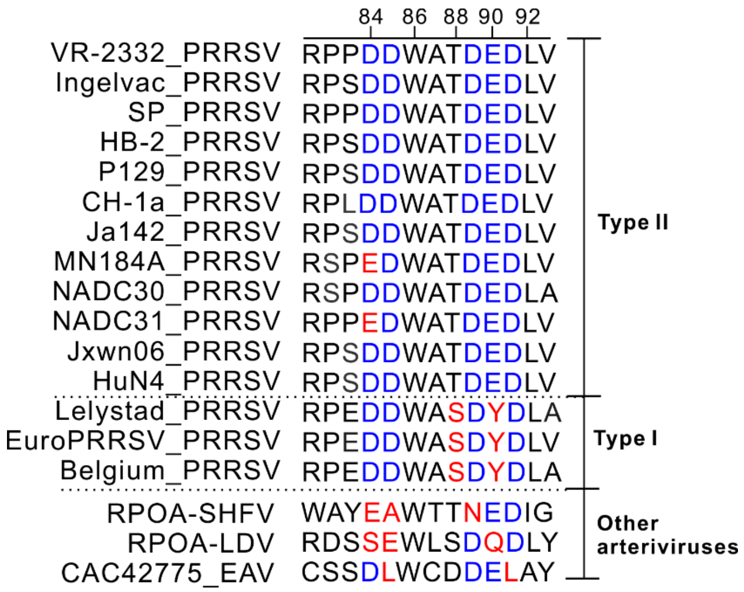

Figure. S1. The sequence alignment of the PLP2 acidic cluster. The amino acids sequences of various PRRSV acidic clusters are presented. The residues investigated in our study are highlighted in red or blue. The accession numbers of viruses are as follows: AY150564 (VR-2332), EF532801 (Igelvac), AF184212 (SP), AY262352 (HB-2), AF494042 (P192), AY032626 (CH-1a), AY424271 (JA142), DQ176019 (MN184A), JN654459 (NADC30), JN660150 (NADC31), EF641008 (JXwn06), EF635006 (HuN4), M96262 (Lelystad), AY366525 (EuroPRRSV), KT159248 (Belgium), Q68722 (RPOA-SHFV), Q06562 (RPOA-LDV), CAC42775 (EAV).
